# Extracts from an Address on Dental Surgery

**Published:** 1872-04

**Authors:** Oliver W. Holmes


### ARTICLE V.

### *Extracts from an Address.*

Delivered at the Commencement Exercises of the Dental Department in Harvard University, February 14, 1872.

BY OLIVER W. HOLMES, M. D.

* * * * A few generalities are all that can be attempted in a discourse like this; enough to give some little idea of what the dental profession has grown out of and what it has grown to; a few hints to make us feel more keenly its importance; a picture or two of old superstitions and fancies and barbarisms to contrast with the enlightened knowledge of our own time; a brief mention of some of the leading modern improvements in the scientific and practical departments relating to the teeth; an explanation of the causes which have kept the dental profession from receiving the recognition it has a right to claim, and a vindication of its title to the regard of the community and to a fair representation of its teachings at the great seats of learning. I mention some of the points I shall touch upon rather than discuss, not under formal headings and with strict adhesion to the order in which I mentioned them, but as they appear to present themselves most naturally.

The great difficulty in handling the subject is the extent of its literature, and the infinite detail of ingenuity which has gone to the bringing about of the perfection of its mechanical processes. The value of the teeth to the human family is so prodigious that so soon as attention was fairly turned to their proper management and the methods of repairing their losses, inventive talent precipitated itself, so to speak, upon the new department of human industry. There is no pearl in any royal crown for which a young queen would give one of her front incisors. And those who know what a perfect organ each one of the teeth is, as shown by the more recent revelations of minute anatomy; what pains nature has taken with its complex organization, will not wonder at the estimate set upon it.

The teeth, in their relation to the beauty of the human countenance, have figured in poetry from the earliest times. "Thy teeth are like a flock of sheep that are even shorn which came up from the washing: whereof every one bear twins and none is barren among them," says the imaginative author of the song of songs. Their whiteness has been compared to that of snow, of Parian marble and of pearls until verse is tired of the images. The ancient poets and satirists are full of allusions to the beauty and deformity depending on the conditions of the teeth. The ladies who made it their business to please, on Bentham's principle of procuring the greatest happiness of the greatest number, had recourse to every kind of artifice to disguise their defects and commend their charms. Here is one of these tricks as given by Athenæus, in a passage not cited by Duval in his large collection of classical quotations relating to this subject. I give a part of it in English.

"They whose teeth are elegant force themselves to smile even against their inclination, so that the beauty of their mouths may be seen by their visitors. But if their smile is not so pleasant a sight, they hold a sprig of myrtle in their mouths so that it will cover their teeth when they open their lips on purpose or otherwise."

As for the unfortunates whose teeth were discolored or had suffered some of the common changes that age brings about, the satirists scoffed at them in such course language that their phrases are quite unbearable to modern ears. Even such a personal remark as that of the graceful Catullus would be considered inadmissible in our time—"Your mouth is full of teeth half a yard long and your gums are like an old wagon-box." The first circumstance—the seeming inordinate length of the teeth—is a well-known effect of age, which produces a shrinking of the gums. The Frenchmen talk of *dents dechaussees*, unshod teeth, and I remember that Thackery, in one of his stories, speaks of certain ladies, not very young ones, as "long in the tooth," among other by no means flattering peculiarities.

We have grown more civil than the Romans, but we know the beauty of a fine set of teeth and the deformity of its opposite as well as they did. It is true that men can often conceal the imperfection of their dental arrangements by letting the eaves of a heavy moustache overshadow their mouths. But to women, to hide whose smile would be to take away half the sunshine of life, and to whom nature has kindly refused the growth that would deprive us of it, there is no element of her wondrous beauty that can take the place of white, even, well-shaped teeth. And as beauty is not a mere plaything, but a great force, like gravity or electricity, the art which keeps it, mends it and to some extent makes it, is of corresponding importance.

But we must add to this the consideration that speech is so largely dependent on the perfection of the teeth that our language, we might almost say, loses a letter with every tooth that falls. What can be more painful to witness than the effort of a hapless friend to bite his consonants out of the alphabet when he is reduced to the condition of an infant, whose boneless gums are unfit for any task but the caressing pressure of the maternal mouthful?

And then the humbler but still necessary function of mastication—how much depends on the ease and perfection with which this is performed? You can tell the state of a village by going to the mill. If it has enough to grind, and grinds it well and cheaply, you will find good farms and well fed people. So if you see a good square jaw filled with good sound teeth, and moved by ten muscles that mean business and do it, you will find in all probability that they nourish a sound frame in man or woman. I have never forgotten the complaint of poor Walter Savage Landor—a sadder one than any of the Preacher's, it seems to me. I quote it from memory. "I have lost my mind," he said; "that I do not care so much about, but I have lost my teeth, and I cannot eat." It has been my custom for many years when lecturing upon this part of anatomy, to bring forward the skull of a large turtle, in order that its jaws might be compared with those of the human being in very advanced years. The sharpened edges of the alveolar border in the old man show the retrograde process by which he returns to the quasi-embryonic condition, reminding us of that earlier period when he passed through the scale of being upward, to reach the supremacy of which age is constantly striving to deprive him.

It is no wonder, then, that the teeth have been particular objects of attention from the earliest period. The Egyptians, who made specialties of everything, had professional dentists, and it is asserted that artificial teeth of ivory or wood, some of them on gold plates, have been found in the jaws of mummies. The teeth of mummies are said also to have been found filled with gold. I do not find any distinct notice of a dental profession from the time of the Egyptians to that of Galen. You will permit me to quote, in the original, a passage which I have unearthed in one of his treatises, because it makes use of an adjective which will be found in our catalogues and diplomas, where it was admitted after some discussion:

Omnes tamen istos communi nomine medicos appellant, perinde ut eos, puto, qui a quibusdam membris, quorum præcipue curam gerunt, vocitantur: hos nanque ocularios, auricularios, DENTARIOS (ita dicere liceat) medicos nominant.

It is a long interval from Galen to the middle of the 17th century. But I have not found any other traces of a special dental profession, until I came upon the following, which looks very much as if it referred to such specialties. In the dairy of the Rev. John Ward, Vicar of Stratford-on-Avon, from 1648 to 1679—the book rendered famous by a reminiscence of Shakspeare, for which the poet would not have thanked him—is the following:

"Uppon a signe about Fleet Bridge this is written—

'Here lives Peter de la Roch and George Goslin, both which, and no other, are sworn operators to the King's teeth.''

Whether these operators had any other calling that this august office does not appear. Early in the next century the practice of dentistry seems te have been in the hands of silversmiths and jewelers. I think many of us can recall the name of a fellow-citizen, originally, I think, a watchmaker, who branched off without any special training into the business of a dentist, and who acquired a considerable name for filling teeth with skill and success, taking time enough about it, and receiving very handsome pay for his services.

Dentistry as a profession may be safely said to have come into existence during the present century. In this country its growth has been of wonderful rapidity. One would have thought that Cadmus had sown a new furrow full of teeth and that they had sprung up dentists. In 1820, it has been computed that there were not more than a hundred dentists in the United States. In 1858 there were four thousand. I presume they have increased in as rapid a ratio since that time, and in the meanwhile works on denistry, journals devoted to it, institution for teaching it, have become so numerous that it is recognized as one of the great callings of life.

If we would know what we have gained by the elevation of dentistry into an honorable special calling, we must go back to the time when ignorance, superstition and bungling awkwardness reigned over the whole province of art, now so fully illuminated by science, and in which such admirable mechanical skill has developed itself in every form to relieve suffering, to supply deficiencies, to add in all possible ways to comfort and comeliness.

It is simply amusing to look back two or three centuries and see what men were capable of believing. You will find in Ambrose Paré various forms of words in use in his time to cure the toothache. The notion that this pain was caused by a worm, which Shakspeare refers to, is at least as old as Avicenda. Strange significance was attached to an anomoly which an old friend of mine told me happened in his own person, I mean the same fact that Richard the Third boasts of, namely, that he was born with teeth. A girl was born at Picenum, with six teeth, says Polydore Virgil; and at that time the Turks began to capture our towns far and wide. But nothing quite equals the story of the miller's little son, whose second molar on the left hand side of the lower jaw was a gold one, as good, they said, as if a goldsmith had made it. Some pretended having seen the letters C S C legibly inscribed upon it. I have before me a most exact statement of the facts of the case, authenticated by a number of eminently respectable personages. And I find in Haller's Bibliotheca Anatomica various notices of the storm of controversy excited by the story of this Silesian boy with the golden tooth. "What it portends," says Laurence Scholtzius, "I do not hesitate to declare is known to God alone." It is to this most famous case that Sir Thomas Brown refers when he says, speaking of the portended difference of posture in which drowned men and women float, "But hereof we cease to discourse, lest we undertake to afford a reason of the golden tooth, that is, to invent or assign a cause, when we remain unsatisfied or unassured of the event." And in the margin, "Of the cause whereof much dispute was made and at last proved an imposture." All this was a good while ago, but I am myself old enough to remember several curious notions about the teeth which had a considerable currency and came near enough to being believed to be told pretty seriously. If one had a tooth extracted, it must be burned, because if a dog got it and swallowed it one would have a dog's tooth come in its place. I recollect a story told me of a somewhat noted public character whose smile or other attractions had made him dangerous to the sex formerly called the weaker one, a personage too well known to the scandal of his time, who was said to have had his teeth, or some of them, extracted and replaced by those of a living animal—a calf or a sheep. This story was told seriously, and the hero of it I have seen with my own eyes, when age had disarmed him of the fatal fascinations of his earlier days. There is a common notion enough still prevalent that some persons have a complete set of double teeth, as they are called; a jaw full of molars. I never saw one, and I doubt if anybody ever did; but the teeth of Indians and sailors, ground down by the attrition of hard grains or sea biscuit, might be mistaken for such a mal-formation.

It is only since the year 1855, that the anatomy of the teeth, out of which necessarily arose new views of their physiology and pathology, can be said to have been fairly understood. It is true that old Leeuwenhoeck had described the "pipes," as he called them, of the dentine so long ago as the year 1678, but his discoveries were so much ahead of his time that they had to wait some generations, like the seven sleepers, before they woke up to find themselves confirmed. An article in the *British and Foreign Medical Review* for the year 1839, brought before the profession in England and America, in a connected way and with illustrative figures, a series of discoveries which had changed the whole aspect of dental anatomy. We owe all these discoveries or re-discoveries to the invention of the achromatic microscope, which enables any of us to show the student the beautiful intricate structure of the teeth as plainly as he can see the anatomy of the skeleton with the naked eye. We have all studied the exquisite tubular arrangement of the dentine, have speculated on the nature of the contents of the tubes, first demonstrated by Owen in the elephant, have examined the prisms of the enamel, and the stellate cells of the cementum; you have seen the vascular systems of the pulp and around the fang and how they run into each other; the tooth is for you a delicately organized, living structure, carrying on nutritive functions through the greater portion of its substance, and capable to a certain extent of repairing its injuries. To those who studied Bell's or Meckel's Anatomy, who read the works of Hunter or Fox on the teeth, all this is a revelation, which only those can fully appreciate who were born in the benighted days of dental heathenism.

While the scientific basis of dental art has made these great advances in modern days, the practice of the art itself has undergone the most wonderful transformation. The work of filling teeth has been carried to such perfection that not only is decay arrested and a tooth which seemed destined to rapid destruction so repaired that it will last a lifetime, but where the greater portion of a tooth was gone, it has been built up, so that the miracle of the boy of Silesia is wrought every day by mortal hands, and we see a golden tooth in a living mouth without fearing an invasion of the Turks, or a war with England. I suppose that the improvements in this particular department of dentistry, the invention and perfecting of mineral teeth, their insertion on plates retained by atmospheric pressure, the substitution of the improved forceps for the clavis, and the application of anæsthesia to extraction, would be considered the greatest achievements of modern dentistry.

What a change since the time when teeth were allowed to decay as if they were not worth the gold it took to fill them. What a change from the time of those ghastly *rateliers*, as the French call them, carved in ivory and supported by springs that creaked with every motion of the jaws like the thoroughbrace of an old-fashioned stage-coach. Could anything be less inviting to social intercourse, could anything be more appalling to tender infancy than the sight of those dancing sets of artificial teeth, looking as if they were ready to jump from their owner's mouths and fasten upon one, as they used to say a turtle's head would do after it was cut off? Mr. Greenwood, of New York, you may remember, carved a set for the Father of his Country, and one can hardly fail to see how the flattened and compressed lips were in a perpetual struggle with those loose fitting arches and rebellious spirals. Yet this was considered a master-piece of dental workmanship, and I have no doubt that pilgrimages have been made to Mount Vernon by artificers in that line of business who left with a tear in one eye at the sight of Washington's majestic conntenance, and a twinkle of satisfaction in the other at the triumph achieved by Mr. Greenwood.

Contrast this state of things with the manufacture of artificial mineral teeth as carried on in this country, where it has been brought to its greatest perfection. More than ten years ago, there were nine factories engaged in their fabrication and more than two million teeth were made in a year. To-day I suppose they must be made and sold by the bushel like the cereal grains, and if the great factories required elevators to handle their products it would hardly surprise us. Compare the delicately tinted, exquisitely shaped porcelain incisors with those frightful ivory palisades that used to play up and down like a portcullis in a manner to terrify all beholders. In fact, the perfection of artificial teeth is carried almost too far. They have come to be for the inside of the head what the wig was for its outside in the days of our ancestors. It was so much more convenient to have a head of hair that one could whisk on and off in a moment, one that never grew gray, one that was just the shade the owner fancied, that was always in curl, that could be laid aside in hot weather to let the cool breeze play over the naked scalp, a luxury which Adam never knew in Paradise, and coming about as near to "sitting in one's bones" as is practicable while we are in the flesh; all this was so much more convenient and comfortable than the arrangement provided by nature, that the wig reigned undisputed for generations, and will, not very improbably, return to bless mankind before our children's children are bald and gray. So with the artificial teeth of this dental millennium in which it is our good fortune to live. They are comely, they never ache, they are contented with their situation and keep their place, which is more than we can say of most of our living servants; they undergo no changes in the mouth, they admit of the nicest personal proprieties, they serve perfectly for articulation, and, though one can hardly crack a peach-stone with them, as some can with their native molars, or use them for biting off the heads of iron nails, as used to be told of Ethan Allen, they can do good service in the respectable and responsible duties of mastication. The consequence of all this is that people are only too ready to have their natural teeth shelled from their gums like so many grains of Indian corn from the cob, and a complete mouthful of artificial make inserted in their place. You miss your friend for a little time—he is in his chamber with his jaws tied up, perusing Zimmermann on Solitude for a few day—suffering from toothache is the figurative language in which his condition is announced. When he returns to society he has recovered his youth, like Æson in the hands of Medea, and his smile is a glittering welcome, a mineral benediction which it is a joy forever to be blest with. Think again what that preliminary process of edentation would have been in the days when the rustic patient complained that he had to pay as much as his neighbor who had been dragged three times round the room before the tooth came out. There never was a claw on bird or beast that was the cause of such anguish of apprehension, such howls of agony as that diabolical instrument, looking like a vulture's talon, but known by the name of "the key." It was a key indeed ; it may have opened the door of heaven to the sufferer in due time, but while the bolt was turning the victim thought he was in that other place, where the man must be who invented the instrument of torture. *Now* a patient comes in, takes a few whiffs of an anæsthetic, has a dozen or more teeth submitted to the embrace of the gentlemanly forceps, which lifts them from their sockets as one takes out the pegs of a solitaire-board; say rather, as a father lifts his first-born infant; comes to ; stares about him; asks when they are going to begin ; is told that it is all over; bursts into tears of hysteric gratitude, kisses the smiling dentist, wants to hug all mankind and make the human race happy at once, sobers down presently, ties up his face, and takes to retirement and Zimmermann for a season, as before mentioned. * * * * * * *

The use of the mallet in filling teeth, every blow of which instrument is a fractional knock on the head to the patient equal to about one hundredth of that which a slayer of cattle gives to a full grown ox to finish him, but which being taken in divided doses allows the sufferer to escape with life. The use of the mallet, automatic or other, far from agreeable as it is, is considered a vast accession to the art of dentistry. "Every man must be anvil or sledge," says Goethe, and it is quite plain that our friends, the dentists have settled it so far as they and we are concerned.

Nothing has excited my admiration more than the wonderful drills moved by the foot or other power which may be preferred, finding their way into every corner of the mouth with a sinuous grace of movement such as the serpent displayed for the facination of our unfortunate first parent, and making their way into the solid dental substance with a rapidity from which the engineers of the Hoosac tunnel might borrow a most significant lesson.

The employment of sponge gold for certain purposes, and the use of heat to develop the cohesive properties of the metal, have enabled the dentist to perform those remarkable feats of building up a tooth from its ruins to which I have before referred.

The public seem hardly to appreciate the great value of the cement filling; both the oxide of zinc and the gutta percha, either of which is capable of preserving for long use a tooth too infirm to bear filling with gold.

I learn that even exposed pulps may be protected by artificial means, and thus a tooth saved as a living organ from an almost hopeless condition.

Important as are these mechanical inventions, the growth of dental associations, educational institutions, and journals, mark a still more general advance of the profession. * * *

The picture of old age drawn in Ecclesiastes is wonderfully impressive, all the more so in consequence of the obscurity of some of its images. But we all know what the Preacher means when he speaks of the drawing nigh of the years when we shall say there is no pleasure in them, and of the day when the grinders shall cease because they are few, and those that look out of the window shall be darkened. There were no dentists in those days to rejuvenate the old man with a third dentition. There were no opticians to supply his vision with the second eyes of old age. The aged people seem to have been in a most forlorn condition at a time when the men of to-day not rarely have a good deal of vitality left and enjoy life and help to make others enjoy it. To us who remember the late Josiah Quincy and Dr. James Jackson, long after they were eighty years old, who knew something by report of lord Lyndhurst and lord Brougham in their later years, it seems strange to hear Barzillai say to king David, "I am this day four score years old: and can I discern between good and evil? can thy servant taste what I eat or what I drink? can I hear the voice of singing men and singing women?"

But what would old age be to a great number of persons without the aid of the dentist and the optician? The worn-out laboring man, unused to books and with limited capacity for social intercourse, may get along well enough perhaps wich his pipe and his seat in the sunshine or by the fireside. Father Abraham may not have felt the need of spectacles; he went to bed early, no doubt; their was no daily newspaper to read, and he did not shave. But what would become of the scholar, or of persons of any cultivation in our days who at fifty or sixty should find themselves cut off from reading, and not improbably rendered unrepresentable, or at least miserably uncomfortably, in society in consequence of imperfect articulation? The care of the eyes is therefore recognized as one of the most important specialties in medicine, and the study of opthalmology has engaged some of the most distinguished professional talent in this country as in Europe. The province of dentistry is only second in importance to the other domain of medical science and art, and rivals it in the intelligence and activity of those who teach and practice it. In one respect it is of greater public interest than the other branch; most children's and young person's teeth require positive attention, whereas their eyes in the great majority of cases take tolerable care of themselves. I think there are twelve times as many dentists in this city as there are oculists. If every one had twenty eyes in the earlier part of his life, and thirty-two when full grown, the numbers of oculists and dentists might be more equal. * * * * *
—*Boston Medical and Surgical Journal.*

---

### SELECTED ARTICLES.

---

